# ESE-1 in Early Development: Approaches for the Future

**DOI:** 10.3389/fcell.2016.00073

**Published:** 2016-06-28

**Authors:** Chan Mi Lee, Jing Wu, Yi Xia, Jim Hu

**Affiliations:** ^1^Program in Physiology and Experimental Medicine, Peter Gilgan Centre for Research and Learning, SickKids Research Institute, SickKids HospitalToronto, ON, Canada; ^2^Laboratory Medicine and Pathobiology, University of TorontoToronto, ON, Canada

**Keywords:** Ets transcription factors, ESE-1, embryogenesis, tissue differentiation

## Abstract

E26 transformation-specific (Ets) family of transcription factors are characterized by the presence of Ets-DNA binding domain and have been found to be highly involved in hematopoiesis and various tissue differentiation. ESE-1, or Elf3 in mice, is a member of epithelium-specific Ets sub-family which is most prominently expressed in epithelial tissues such as the gut, mammary gland, and lung. The role of ESE-1 during embryogenesis had long been alluded from 30% fetal lethality in homozygous knockout mice and its high expression in preimplantation mouse embryos, but there has been no in-depth of analysis of ESE-1 function in early development. With improved proteomics, gene editing tools and increasing knowledge of ESE-1 function in adult tissues, we hereby propose future research directions for the study of ESE-1 in embryogenesis, including studying its regulation at the protein level and at the protein family level, as well as better defining the developmental phase under investigation. Understanding the role of ESE-1 in early development will provide new insights into its involvement in tissue regeneration and cancer, as well as how it functions with other Ets factors as a protein family.

## Introduction

E26 transformation-specific (Ets) transcription factors are characterized by the presence of conserved Ets-DNA binding domain which recognizes a core sequence of GGAA/T, consisting a protein family of at least 27 members in human and 26 in mice (Bult et al., 2008; Hollenhorst et al., 2011). Numerous studies in cell lines and gene disruption animal models have identified function of individual ETS factors as well as their evolutionary relationships, which are reviewed elsewhere (Bartel et al., 2000; Sharrocks, 2001; Oikawa and Yamada, 2003; Gutierrez-Hartmann et al., 2007). However, most studies have focused on single-protein analysis or *in vitro* DNA binding assays that do not reveal Ets proteins acting *in vivo* (Sementchenko and Watson, 2000), when global analyses of Ets factors have revealed that over 16 of Ets members are co-expressed in a given cell type and more than 8 ubiquitously so (Hollenhorst et al., 2004). The potential Ets targets can extensively overlap (Hollenhorst et al., 2007) due to the conservation of mode and specificity of the Ets-DNA binding domain (Sharrocks, 2001; Wei et al., 2010).

Ets factors have been divided into four sub-groups by difference in preferred Ets-binding motifs, which were determined by variations in amino-acids that interact with the backbone of the core recognition sequence (Wei et al., 2010). Nevertheless, there is still redundancy within sub-groups with factors binding to the same cognition signal. Class I factors, for example, include at least 11 Ets factors and mainly recognize CCGGAA/T, while Class III factors, which include ESE-1 and at least 5 others, demonstrate strong preference for GCGGAAC (Wei et al., 2010). Therefore, other determinants of specificity, such as protein-protein interactions with other transcription factors via distinct protein domains and additional regulatory sequences flanking the core Ets-binding site on target genes, are as important to recognize how Ets factors function as a family within a cell. This means that manipulation of a single Ets factor may result in a mitigated phenotype due to functional compensation from other members. This particularly applies to embryogenesis, where some Ets factors have been shown to be redundant (Kageyama et al., 2006). Therefore, more holistic approaches are required to understand the interplay of Ets factors during early development.

In this Perspective, we focus on a well-characterized member of a subfamily of Ets factors called ESE-1, or Epithelium-specific Ets transcription factor 1, to provide an overall direction of future research on ESE-1 and other Ets factors in fetal development. ESE-1 homolog in mice is known as Elf3. Epithelial-specific Ets factors, or ESEs, constitute a subfamily of Ets factors which include ESE-1/Elf3, ESE-2/Elf5, ESE-3/Ehf, and PDEF, whose expression patterns are known to be mostly restricted to epithelial tissues (Sharrocks, 2001). The role of ESE-1 is more thoroughly reviewed elsewhere (Oliver et al., 2012), and has been studied primarily in epithelium differentiation. ESE-1 therefore has received less attention regarding its role in early development, despite evidence suggesting its involvement as discussed below. With recent advances in proteomics and genomics as well as gene editing tools such as CRISPR/Cas, it is time to build more knowledge on how ESE-1 is modulated at protein level and how it functions in combination with other Ets factors during embryogenesis. Analysis of Ets intra-family interaction and regulation during embryogenesis will provide better insight into other protein families that are likewise comprised of multiple and redundant members such as HOX, GATA, and FOX proteins which share highly similar DNA-binding domains (Messina et al., 2004), as well as providing functional insight into the role of ESE-1 in cancer and tissue regeneration.

## The role of ESE-1 in embryonic development

The involvement of ESE-1 in early development was first identified in the diminished survival of *Elf3* homozygous knockout mice, where targeted deletion of *Elf3* resulted in 30% of fetal lethality at around embryonic day 11.5 (E11.5) (Ng et al., 2002). However, no gross histopathological anomalies were found to explain the cause of death (Ng et al., 2002). Similarly, disruption of *Elf3* by siRNA injection reduced embryo survival by 50%, while co-suppression of three prominent Ets factors expressed in mouse preimplantation embryos *Elf3, Etsrp71*, and *Spic* blocked development to blastocysts by 70%, involving reduction of key regulatory genes *eIF-1A* and *Oct3/4* that contain Ets-binding sites (EBS) in their promoters (Kageyama et al., 2006). Recent reports have also shown inhibitory effect of ESE-1 on stem cell transcription factor *Oct4* expression by binding to EBS in the conserved region 2 (CR2) of the *Oct4* promoter during retinoic acid (RA)-induced differentiation of human embryonic carcinoma cell line NCCIT (Park et al., 2014). *Oct4* is essential in the maintenance of stem cell pluripotency and its loss of expression leads to cell differentiation (Pan et al., 2002). Therefore, the inverse relationship between *Elf3* and *Oct4* and the close involvement of *ESE-1* in epithelial cell differentiation in various tissue types such as skin, small intestine and mammary gland may indicate that *ESE-1* is a marker of epithelial tissue development. However, the exact gene targets or functional mechanism of *ESE-1/Elf3* during early development have not been elucidated.

## Regulation of ESE-1 expression

Promoter analysis of *ESE-1* has revealed four main regulatory regions Ets, CAAT, TATA, and NFκB (Oettgen et al., 1999). However, different regions of the upstream segments of *ESE-1* gene may be more critical than others during development and differentiation. For example, Hou et al. demonstrated that in retinoic acid-differentiated murine embryonal carcinoma (EC) cell line F9, there is a substantial increase in the utilization of an upstream regulator region approximately 2 kb upstream of the transcription start site that was responsible for the increase in the *ESE-1* promoter site (Hou et al., 2004). While it is well-known that long-range enhancers have important role in mammalian gene regulation (Heintzman and Ren, 2009), factors which bind to the enhancer region of *ESE-1* gene have not been well-defined. A conserved 30 base pair (bp) *ESE-1* enhancer sequence (ESS) was also shown to be essential in regulating *ESE-1* transcription in response to epithelial differentiation signals, controlled by two ESS-binding protein complexes that are of uncertain identity (Neve et al., 2006). In mouse embryo, *elf3* has been shown to be expressed at low levels in mouse meiosis II (MII) oocytes, and increase at one-cell stage embryo, briefly decline at the two-cell stage, and increase again until the blastocyst stage (Kageyama et al., 2006). Therefore, more investigation is needed to elucidate the *ESE-1* enhancer elements and their binding nuclear factors, as well as signals which activate *ESE-1* gene transcription during embryogenesis. The most notable regulator of *ESE-1* so far known is NFκB, which is required for *ESE-1* induction in response to pro-inflammatory cytokines (Rudders et al., 2001; Wu et al., 2008). Whether this applies to embryogenesis, however, is unknown. Additionally, ESE-1 has been shown to downregulate its own cytokine-induced expression (Wu et al., 2008), possibly by auto-inhibition through intramolecular interaction between the N-terminal transactivation domain and the C-terminal ETS domain (Kopp et al., 2007). Other non-transcriptional mechanisms of regulation of *ESE-1*, such as through microRNAs (Di et al., 2014; Qin et al., 2015) has never been explored. Modern molecular techniques such as long range interactions (4C experiments) (Cai et al., 2016) or knock-down of specific transcription factors motifs on the promotor and enhancers of ESE-1 using CRISPR/Cas9 may reveal interesting information about ESE-1 regulation in the future.

## Building an overall picture and approaches for the future

As mentioned above, there is dearth of information on the exact role of ESE-1 during embryogenesis, despite evidence suggesting its involvement during blastocyst formation and organogenesis. We therefore outline three main future research objectives for the study of ESE-1 function during early development:

### Better analysis of ESE-1 function at the protein level

**Figure 1:**
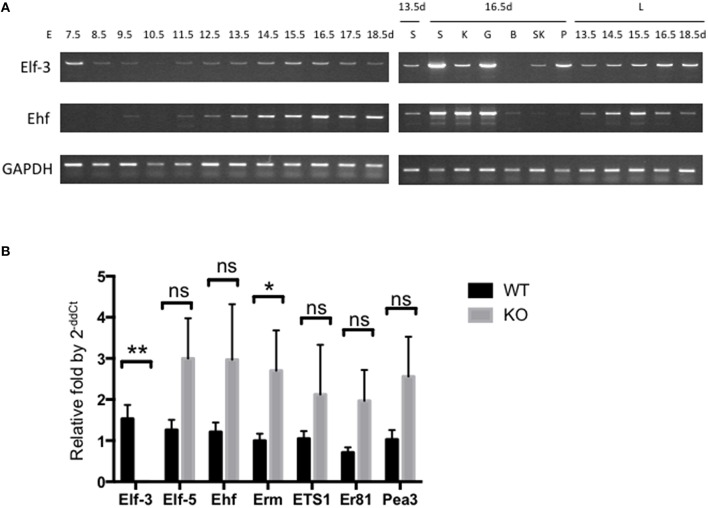
**Expression of ***ESE-1/Elf3*** and ***ESE-3/Ehf*** during mouse embryogenesis. (A)** Limited cycle RT-PCR analysis of *Elf3* and *Ehf* gene expression. Total mRNA from whole mouse embryo were collected at indicated time points of development **(Left Panel)**, as well as from individual organs **(Right Panel)** such as lung (L), salivary gland (S), kidney (K), gut (G), brain (B), skeletal muscle (SK), and Pancreas (P). The isolated mRNA were subjected to limited cycle RT-PCR analysis across different time points ranging from Embryonic Day (E) 7.5–18.5. GAPDH gene was included as an internal control. RT-PCR products from each data point were loaded onto agarose gel to visualize their size and intensity. Six to eight embryos were collected at early data points (E7.5-E10.5), and data shows *n* > 3 for each data point. **(B)** RT-qPCR of various Ets factors in wild-type and *Elf3*^−∕−^ adult mouse lung. The graph shows *n* = 4–6 per data point, and statistical analysis was done by two-tailed Student *t*-test with Welch's correction where appropriate. “ns” indicates not significant; ^*^ and ^**^ indicates *P* < 0.05 and *P* < 0.01, respectively.

Most expression analysis on parts of mouse embryo for the expression of *ESE-/elf3* and *ESE-3*/*ehf* in different tissues have so far focused on quantifying levels of mRNA, by Northern blot or RT-PCR of isolated tissues and cell lines. Since there has not been a longitudinal analysis of expression during mouse embryonic development, we assessed the mRNA levels of *ESE-1/Elf3* and *ESE-3/Ehf*, whose Ets domain is 84% identical to ESE-1 Ets domain (Kas et al., 2000), in wild-type (WT) mice during E7.5-E18.5 by limited cycle RT-PCR as a preliminary analysis (Figure 1A). Albeit with varying degrees, we found that most of the *Elf3* and *Ehf* expression occurred between E13.5 and E18.5 during active organogenesis and tissue differentiation and in E7.5 following gastrulation (Loebel et al., 2003; Tam and Loebel, 2007; Takaoka and Hamada, 2012). Further analysis by quantitative RT-PCR may help confirm and quantify these expression levels. However, RNA-based studies are limited in that they identify transcriptional changes that are regulated both directly and indirectly (Sementchenko and Watson, 2000), and that they do not reveal protein modulations which may be more functionally relevant, by mechanisms such as protein-protein interactions and post-translational modifications that alter protein activity or stability. It is understandable that there has been lack of high quality antibodies which could recognize Ets factors of interest in the past. However, with increased availability of better ESE-1 antibodies and proteomic tools (Sydor and Nock, 2003; Pauly et al., 2013), it is time to delve into studying the expression and function of ESE-1/Elf3 at protein level.

One of the important determinants of Ets specificity is tissue expression pattern (Hollenhorst et al., 2004; Wei et al., 2010). However, there has been no comprehensive study on the localization of Ets proteins during development, and known facts on ESE-1 expression have been derived from Northern blot analysis on human fetal tissues (Oettgen et al., 1997), and E19 mouse embryo tissues (Tymms et al., 1997). Therefore, immunohistochemistry of Elf3 in fixed mouse fetus over the time course of development may be a good starting point to get better understanding of ESE-1 protein expression and its tissue distribution pattern. Understanding protein expression and modulation will provide stepping stones to ascertaining ESE-1 function and regulation at the protein level. Additionally, Ets factors are known to undergo various post-translational modifications such as phosphorylation, glycosylation, ubiquitination, sumoylation, and acetylation (Tootle and Rebay, 2005). Post-translational modification can influence protein activity, protein-protein interactions, protein-DNA interactions, subcellular localization, and stability, thus significantly increasing the functional versatility of a protein (Tootle and Rebay, 2005). The most promising post-translational modification in ESE-1 is phosphorylation, where ESE-1 has previously been shown to be stabilized by serine phosphorylation, which altered its subcellular localization and protein half-life in breast cancer (Manavathi et al., 2007). NetPhos2.0 server predicts 15 potential serine phosphorylation sites on ESE-1 protein with P-score of >0.95. Thus, whether ESE-1 is post-translationally modified during embryogenesis should be further investigated, and the development of phospho-ESE-1 antibody may help in functional analysis of phosphorylated ESE-1.

ESE-1/Elf3 is also unique to the family of Ets factors that it contains two AT-hook domains which can potentially enable ESE-1 to bind in the minor groove of AT-rich DNA and interact with other proteins (Kopp et al., 2007). Protein binding partners have been shown to be critical in regulating Ets factor function (reviewed in Li et al., 2000), but there is limited literature on ESE-1 binding partners, only identified through GST-pull down assays or immunoprecipitations in cell lines such as endothelial cells and 293T cell line (Rudders et al., 2001; Longoni et al., 2013). Therefore following identification and characterization of ESE-1/Elf3 protein expression during embryogenesis, mass spectrometric analysis of embryonic proteins (Nagano et al., 2005) altered in *Elf3*^−∕−^ embryonic stem cells (ES) and co-immunoprecipitated binding partners in WT mouse ES nuclear extract (Liang et al., 2008) will provide great insights into its function during early development through protein interaction.

### Better analysis of ESE-1 function as a member of the Ets protein family

Identification Ets target genes has mostly been derived from gain or loss of function studies. Single knockout animal models have been useful in revealing function of individual Ets factors by distinct phenotypes (Bartel et al., 2000). However, there has been virtually no in-depth analysis of changes in other Ets factor expression or post-translational modifications, while studies indicate that over two-thirds of Ets family members are co-expressed in most cell types (Hollenhorst et al., 2004). Notably, Ets factors are expressed temporally during differentiation, showing sharp transitions in expression that correlate with critical commitment (Anderson et al., 1999) or growth events (Bhat et al., 1987), and distinct pattern of co-expression during tumorigenesis (Galang et al., 2004). Therefore, understanding this combinatorial nature of Ets family members is essential in identifying the physiological function of Ets factors *in vivo*.

Ets factors are known to physically and functionally interact with each other, such as the binding between Tel and Fli-1 (Kwiatkowski et al., 1998) and between Ets2 with Ets1 and Erg (Basuyaux et al., 1997). Analysis of other Ets factors in *Elf3* KO mouse lungs also revealed upregulation of other Ets factors in the absence of *Elf3*, notably *Erm* (Figure 1B). One approach to studying the interplay between other Ets factors, therefore, would be to examine changes in expression and protein activity of other Ets factors in homozygous knockout mice of a single or double Ets gene(s), thus identifying possible source of compensation from other Ets family members. High-throughput analyses of transcriptional binding specificities have already been shown to be more physiologically relevant than single-protein studies, as exemplified by comparison study of homeobox protein family (Wei et al., 2010). This similarly applies to revealing tissue-specific interactions of Ets proteins, where examples of Ets family interaction have shown tissue-dependent functional redundancy of Ets factors. For example, in *Ets2* knockout mice, Ets1 along with other gene targets such as *MMP3, MMP9*, and *uPA* are reduced in skin but unchanged in mammary gland (Yamamoto et al., 1998). Protein microarrays are available for tissue-specific interactions (Poetz et al., 2005; Stoll et al., 2005; Dominguez et al., 2008), and initial analyses of tissue expression pattern would be critical to guide this investigation.

**Figure 2:**
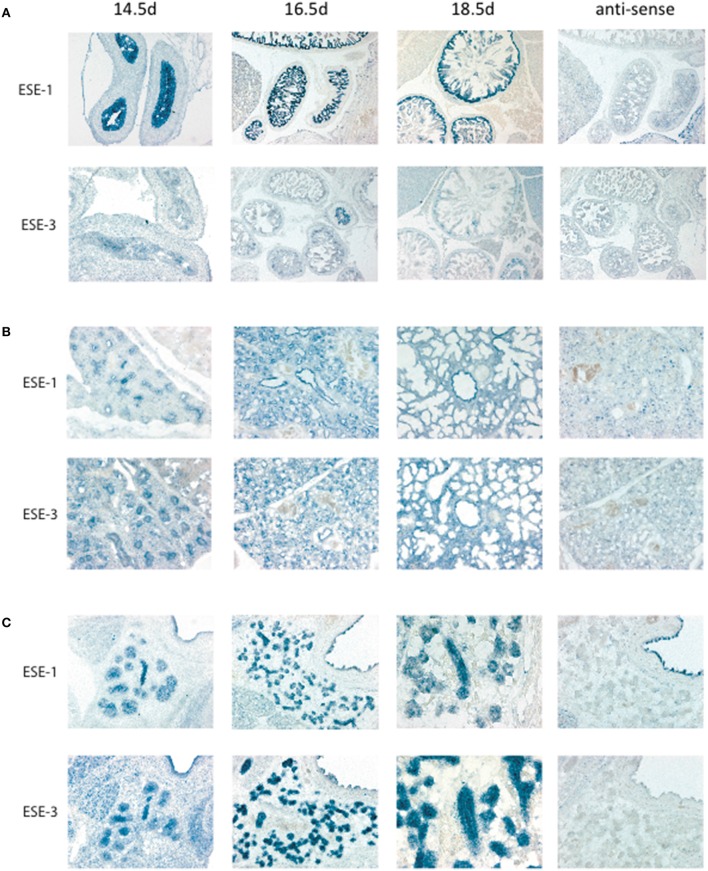
*****In situ*** hybridization of ***Elf3*** and ***Ehf*** for tissue localization during development**. *In situ* hybridization was performed as previously described in Hui and Joyner (1993). Mouse *Elf3* full length cDNA plasmid was used as template for the digoxygenin-labeled RNA probe. *Elf3* and *Ehf* were detected in **(A)** Gut, **(B)** Lung, and **(C)** Salivary gland with anti-sense probe as a negative control. Pictures were taken at different time points from Embryonic Day (E) 14.5–18.5. Figure shows a representative result from *n* > 3 mice for each data point.

For ESE subfamily, *ESE-1* and *ESE-3* are known to be expressed mostly in the epithelium in both fetal and adult tissues (Kas et al., 2000; Tugores et al., 2001; Silverman et al., 2002), and *ESE-1* has been shown to upregulate *ESE-3* during inflammation (Wu et al., 2008). To study whether *ESE-1* and *ESE-3* expression therefore overlap during embryogenesis, we performed *in situ* hybridization of the gut, lungs, and salivary glands as representative tissues for *ESE-1* and *ESE-3* expression. Interestingly, we found a differential pattern of expression in the two *ESEs* where *ESE-1* was more predominantly expressed in the gut compared to *ESE-3* (Figure 2A), and *ESE-3* being slightly more robust in the lungs (Figure 2B), and both *ESE's* were present at similar levels in the salivary gland (Figure 2C). Therefore, conclusions drawn from one study using different cell type and condition may not apply to other tissue models, and specific definition of experimental context is vital to study Ets factors during early development as discussed further below.

### Better definition of the physiological context

Embryogenesis and fetal development consist of multiple steps which involve rapid changes in cellular symmetry, position, survival, and differentiation at the whole organismal level. The microenvironment of a cell, such as stem cell niche, has a critical impact on the cellular developmental potential and differentiation (Martinez-Agosto et al., 2007; Lane et al., 2014). Therefore, precise definition of the developmental phase of interest is important to more accurately represent the particular *in vivo* microenvironment *in vitro*. Determination of specific time point and tissue context during development, instead of generic differentiation models using embryonic carcinoma cell lines (Hou et al., 2004; Park et al., 2014), will thus be more informative in designing *in vitro* models representative of the developmental stage of interest to study the role of *ESE-1* during embryogenesis, and relate the information back to the *in vivo* setting.

Previous findings on *ESE-1* provide hints to the most promising time points and tissue settings to focus on. Hierarchical clustering of the transcriptome over the time course of embryonic development, for example, revealed that E11.5-E12.0, during which *Elf3*^−∕−^ embryos show partial fetal lethality, is associated with dramatic induction of genes involved in adult hemoglobin production and erythrocyte differentiation (Wagner et al., 2005). The transcription of the beta hemoglobin chain starts at E12.0, including other genes involved in hemoglobin biosynthesis such as *erythroid associated differentiation factor (ERAF)*, and *heme binding protein 1 (HEBP1)* (Wagner et al., 2005). Also, E11.5-E12.0 is the critical time point where definitive hematopoiesis begins in the liver (Wagner et al., 2005). Therefore, defects in hemoglobin synthesis may have been partially responsible for embryonic lethality in *Elf3*^−∕−^ mice, and studying the effect of *ESE-1* deletion by genome editing (Peters et al., 2008; Musunuru, 2013) in *in vitro* models of erythropoiesis such as human inducible pluripotent stem cells (iPSCs) (Malik et al., 1998; Lapillonne et al., 2010; Kobari et al., 2012) may help to identify contribution of *ESE-1* in embryonic hemoglobin synthesis.

The involvement of *ESE-1* in lung development may similarly provide a good model, given distinct pattern of expression of *ESE-1* in the human fetal lung vs. the adult tissue, the availability of *in vitro* models in 3D cultures to study branching morphogenesis from embryonic lung explants (Warburton et al., 2005; Del Moral and Warburton, 2010), and well-characterization of signaling pathways involved in lung development such as FGF10 and SHH signaling (Iber and Menshykau, 2013), which are the two most important regulatory diffusible proteins that facilitate outgrowth of lung buds and branching. One way to test whether *Erm*, an Ets factor which was highly expressed in *Elf3*^−∕−^ lung (Figure 1B), functionally compensate for the loss of *Elf3* would be to generate *Elf3*^−∕−^*Erm*^−∕−^ double knockout and investigate the fetal lung development. However, while mice have been used as the primary model for the study of mammalian development given their genetic similarity to humans, short generation time, and the ease of gene manipulation, the human and mouse airways are anatomically very different. For example, basal cells which are limited to trachea in mice are present throughout the human airways (Rock et al., 2009; Rock and Hogan, 2011), and Clara cells which are confined mostly to distal bronchiole airways in humans are found throughout the murine airways (Boers et al., 1999; Rawlins et al., 2009). Therefore, another significant advantage of studying lung morphogenesis would be the availability of human cell models of 3D lung morphogenesis using immortalized human bronchial epithelial cells (Franzdottir et al., 2010; Kaisani et al., 2014), which have been shown to be multipotent (Delgado et al., 2011), to complement the animal studies.

## Concluding remarks

The role of *ESE-1* during embryogenesis had long been implicated in previous discoveries, but there has been no in-depth analysis of *ESE-1* function in early development. With improved proteomics and gene editing tools that are now available, therefore, future studies which elucidate the role of *ESE-1* during embryonic development will provide not only better insights into ESE-1 function in specific cell differentiation pathways, but also its interactions with other Ets family members. In addition, understanding how epithelium-specific Ets factors contribute to normal embryonic development by, for example, global gene expression analysis in systemic knockdown study (Atabakhsh et al., 2012), will help us to better understand how they take part in disorders and abnormalities which share signaling pathways and gene clusters involved in embryogenesis, such as cancer (Monk and Holding, 2001; Kim and Orkin, 2011; Atabakhsh et al., 2012; Smith and Sturmey, 2013) and tissue regeneration (Poss, 2010). This may open new opportunities to tackle aberrant cell differentiation or repair mechanisms that pose barriers to effective treatment, while building on our current body of knowledge on how multi-member protein families function collectively.

## Author contributions

CL and JW contributed equally to the manuscript. CL critically evaluated the data and wrote the manuscript. WJ designed the experiments and performed embryo analysis. YX performed data analysis and finalized the figures. JH supervised the project and critically reviewed the manuscript.

### Conflict of interest statement

The authors declare that the research was conducted in the absence of any commercial or financial relationships that could be construed as a potential conflict of interest.
